# Emotional pictures and time: The effects of arousal and valence on the perception of duration and the subjective passage of time

**DOI:** 10.3758/s13414-026-03241-8

**Published:** 2026-03-24

**Authors:** Daniel Bratzke

**Affiliations:** Department of Psychology, University of Bremen, Bremen, Germany

**Keywords:** Time perception, Emotion, OASIS, Passage of time

## Abstract

Previous studies have shown that emotional stimuli, such as pictures and sounds, can affect the perception of time. The present study investigated the effects of arousal and valence of standardized emotional pictures on two different subjective experiences of time: duration and the passage of time (POT). Emotional pictures were presented for 2, 4, or 6 s, and after each presentation participants provided either a duration or a POT judgment (in different experimental halves) as well as valence and arousal ratings on visual analog scales. In contrast to previous findings, the results showed no effects of arousal and valence on duration judgments; however, there were significant effects of arousal and valence on POT judgments, with both higher arousal and valence leading to an accelerated POT. The absence of arousal and valence effects on duration judgments in the present study could be related to the fact that pictures with extreme content were not included and thus the entire spectrum of possible arousal and valence values was not tested. Importantly, however, the different patterns of result for duration and POT judgments align with a growing body of research indicating that the perception of duration and the experience of the POT are not necessarily directly linked. Furthermore, the present study highlights the importance of collecting and analyzing current affective ratings even when using standardized emotional pictures, as the current ratings differed significantly from the norm values of the standardized picture sets.

## Introduction

Emotions can shape our perception of time. Traditionally, research on emotion and time perception has focused on the perception of duration (for reviews, see Droit-Volet & Meck, 2007; Lake et al., 2016). Since the turn of the century, however, there has also been an increasing number of studies on the relationship between emotional states and the feeling of the passage of time (POT; for reviews, see Droit-Volet & Martinelli, 2023; Wearden, 2015). This research was partly inspired by earlier research on the experience of “flow” (a feeling of timelessness; Csikszentmihalyi, 1988; see, e.g., Droit-Volet & Martinelli, 2023; Larson & Eye, 2006) and the common experience that time seems to pass more quickly as we get older (e.g., Droit-Volet & Wearden, 2015; Wittmann et al., 2015; see Wearden, 2015). In studies that considered both types of judgments, a rather weak relationship between duration and POT judgments was observed, especially for short durations in the seconds range (e.g., Droit-Volet et al., 2017; Droit-Volet & Wearden, 2016; Martinelli & Droit-Volet, 2022a), suggesting that distinct mechanisms underlie these two different temporal experiences (see also Lamprou-Kokolaki et al., 2024).

Duration perception is commonly explained by internal clock models (e.g., Gibbon et al., 1984; Zakay & Block, 1997), which assume that a pacemaker elicits pulses (counted by an accumulator) and the counted pulses represent the to-be-timed duration. The perceived duration of a given interval can vary due to differences in arousal (Treisman, 1963), with higher arousal leading to a higher pacemaker rate and thus longer perceived duration, and also due to attentional allocation, with less attention to temporal information leading to a loss of pulses and thus shorter perceived duration (e.g., Zakay & Block, 1997). Consistent with these assumptions, arousal and attention are usually considered to play crucial roles in the influence of emotion on duration perception (Droit-Volet & Meck, 2007; Lake et al., 2016). In contrast, the feeling of POT has been assumed to be based on the introspective analysis of internal states (e.g., emotions), which result from the effect of internal and external contextual changes on the minimal self (e.g., Droit-Volet & Martinelli, 2023; Martinelli & Droit-Volet, 2022b). The relationship between these two theoretical accounts is largely unclear. However, Martinelli and Droit-Volet (2022b; see also Martinelli & Droit-Volet, 2022a) suggested that in prospective timing paradigms (i.e., when participants know in advance that they are asked for temporal judgments), POT judgments can be based on duration estimation, when changes in stimulus duration are the most salient information. Apart from that, activity-related attention (e.g., intellectual engagement) and hedonic valence (as e.g., in boredom) seem to be especially important factors for the experience of the POT (e.g., Droit-Volet & Wearden, 2016; Wearden, 2015; Martinelli & Droit-Volet, 2022a).

One way to study the influence of emotion on time perception is to evoke temporary emotional states by letting participants view or listen to emotional stimuli. In one of the first systematic studies on the effects of emotional pictures on perceived duration, Angrilli et al. (1997) observed an interaction between the two affective dimensions arousal and valence, with longer perceived duration for high-arousal positive and low-arousal negative pictures than for high-arousal negative and low-arousal positive pictures. They explained this interaction pattern by assuming the coexistence of two different motivational mechanisms, one emotion-driven (active in high-arousal situations) and the other attention-driven (active in low-arousal situations). Accordingly, for high arousal pictures, negative pictures evoke an avoidance reaction (leading to overestimation) and positive ones evoke an approaching reaction (leading to underestimation). For low-arousal pictures, however, negative pictures induce more information processing than positive ones, and therefore less attentional resources are available for the processing of temporal information for negative pictures (leading to relative underestimation). The interaction pattern of Angrilli et al. was replicated by Smith et al. (2011), but only for relatively long durations from 400 to 1,600 ms (Angrilli et al., 1997, used 2, 4, and 6 s) and not for short durations below 300 ms. Van Volkinburg and Balsam (2014), however, could not replicate Angrilli et al.’s interaction pattern using durations of 0.8 and 3.5 s. Instead, they observed that duration estimates increased with increasing arousal, while they were unaffected by valence. Similarly, Noulhiane et al. (2007) could also not replicate the interaction pattern of Angrilli et al. with auditory stimuli using the same durations as Angrilli et al. In their study, negative sounds were judged to be longer than positive ones and high-arousal sounds were judged to be shorter than low-arousal sounds. They suggested that while arousing stimuli may accelerate the pacemaker rate of an internal clock (which should result in longer perceived duration), they are also particularly effective at capturing attention, which could lead to a loss of clock pulses (resulting in shorter perceived duration).

Thus, the same attentional mechanism (in terms of divided attention between stimulus and temporal information) has been put forward to explain completely different results, the relative underestimation of positive compared with low-arousal negative pictures in Angrilli et al. (1997) as well as the relative underestimation of high arousal compared to low arousal stimuli in Noulhiane et al. (2007). It is important to note that these different results cannot be attributed to differences in the tested durations or timing method, as the same durations (2, 4, and 6 s) and timing method (temporal reproduction)^1^ were used in both studies. Taken together, the influence of emotional stimuli, and more specifically their associated arousal and valence, on perceived duration is far from clear. It appears that both arousal and valence can influence the perceived duration, but exactly what these influences are seems to depend on the specific study and the stimulus modality used. Similarly, in their comprehensive meta-analytical review on emotion and time perception, Cui et al. (2023) identified large inconsistencies regarding the effects of valence and arousal on duration perception. Nevertheless, their analysis revealed that negative valence tends to result in longer perceived duration than positive valence, that increasing arousal leads to a prolonged perceived duration, and that scenic pictures, facial expressions and sounds are more effective than words in influencing duration perception. Furthermore, estimation appears to be a more effective method of measuring the effects of emotional stimuli on duration perception than temporal reproduction (e.g., via a continuous key press).

To my knowledge, there is only one study so far that has investigated the influence of emotional pictures on the POT experience. In this study, Martinelli and Droit-Volet (2022b) showed their participants videos containing picture sets that differed in emotional valence (negative, neutral, and positive) for durations ranging from 22 to 123 s and assessed POT judgments. They observed that the speed of the POT slowed down with increasing duration (see also Bratzke, 2025; Jording et al., 2022; Lamprou-Kokolaki et al., 2024), as well as with negative compared to positive valence of the pictures. These results regarding emotional valence are in line with other research suggesting that negative affect leads to a decelerated POT (e.g., Droit-Volet & Wearden, 2015; Martinelli & Droit-Volet, 2022a). These authors, however, did not control for arousal, so the role of arousal as a potential confound in their particular study and also in the influence of emotion on POT judgments in general remains unclear.

The present study aimed at investigating the effects of arousal and valence of emotional pictures on POT as well as duration judgments. Similar to the study design by Angrilli et al. (1997), participants were presented with emotional pictures for different durations and provided temporal judgments (duration and POT) as well as arousal and valence ratings after each picture. The same durations as in Angrilli et al. were used (2, 4, and 6 s; see also Noulhiane et al., 2007). These are in the time range (seconds range), in which dissociations between duration and POT judgments have been previously observed (e.g., Droit-Volet & Wearden, 2016). The pictures were taken from the Open Affective Standardized Image Set (OASIS; Kurdi et al., 2017) instead of the International Picture System (IAPS; Lang et al., 1997). The pictures from OASIS are not subject to the copyright restrictions that apply to IAPS and therefore can be used in online studies without restrictions. The two different temporal judgments were assessed in different halves of the experiment. Based on internal clock models, one would expect that arousing pictures lead to longer duration judgments, as more arousal should speed up the pacemaker rate (Treisman, 1963). Alternatively, the processing of high-arousal as well as low-arousal negative pictures might consume attentional resources (Noulhiane et al., 2007; see also Droit-Volet & Meck, 2007), which could lead to a loss of pulses and therefore a relative underestimation of the duration of these pictures. Based on previous results, one would expect the subjective POT to slow down with increasing duration and also to be slower for negative pictures than for positive ones (Martinelli & Droit-Volet, 2022b).

## Method

### Participants

Ninety-four volunteers participated for course credit. The data of seven participants were excluded because of missing data. The final sample thus consisted of 87 participants with a mean age of 25.8 years (*SD* = 5.5; 68 women, 19 men). Power analyzes (with power =.80 and alpha =.05) yielded a required sample size of at least *N* = 28 for the interaction between arousal and valence on duration estimates reported by Angrilli et al. (1997), and a sample size of *N* = 81 for the effect of valence on POT judgments reported by Martinelli and Droit-Volet (2022b). Thus, the final sample size of 87 participants ensured a statical power of at least .80 for the effect sizes observed in these previous studies. All participants provided informed consent prior to data collection. All procedures performed in the present study were in accordance with the ethical standards of the institutional and/or national research committee and with the 1964 Helsinki declaration and its later amendments or comparable ethical standards. Before the study, participants were informed that they would see pictures that could trigger emotions, including unpleasant ones.

### Apparatus and stimuli

The experiment was an online experiment and run on the participant’s individual computer. It was created in PsychoPy (Peirce et al., 2019) and hosted by Pavlovia (https://pavlovia.org). The stimuli were 24 pictures from OASIS (Kurdi et al., 2017). Pictures displaying explicit pornographic content or humiliated bodies (human or animal) were not included for ethical reasons. The included pictures were selected to form four distinct clusters in terms of all combinations of low vs. high arousal and low vs. high valence. Specifically, the pictures with the IDs 256 (Dog 6), 527 (Nude couple 1), 531 (Nude couple 5), 535 (Nude couple 9), 538 (Nude couple 12), and 661 (Rainbow 2) formed the high arousal/high valence subset (mean arousal: 5.1, mean valence: 5.7), with the IDs 303 (Explosion 2), 871 (War 8), 864 (War 1), 328 (Fire 11), 452 (KKK rally 2), and 857 (Volcano 2) formed the high arousal/low valence subset (mean arousal: 5.1, mean valence: 1.9), with the IDs 162 (Clean 1), 290 (Eating 3), 401 (Grass 3), 402 (Grass 4), 634 (Pinecone 3), and 651 (Pumpkin 1) formed the low arousal/high valence subset (mean arousal: 2.3, mean valence: 5.1), and with the IDs 88 (Bored pose 3), 379 (Garbage dump 1), 385 (Garbage dump 7), 749 (Sidewalk 6), 887 (Windmill 1), and 891 (Yarn 1) formed the low-arousal/low-valence subset (mean arousal: 2.4, mean valence: 2.8). The pictures with the IDs 624 (Pig 1) and 392 (Gazing 4) were used for attention check, and the picture with the ID 860 (Wall 2) was used as background.

### Tasks and procedure

In each trial, first the background picture was shown for a random interval between 5 and 10 s, together with the written instruction to relax. Then, one of the emotional pictures appeared for one of three possible durations (2, 4, or 6 s). After the offset of the picture, participants were first asked to estimate the duration of the picture (or report their POT experience) on a visual analog scale (VAS), ranging from 0 to 10 s (or from “very slowly” to “very fast” in the case of POT judgments). After that, a new screen appeared with two VASs, one for arousal with the poles “calm” (1) and “aroused” (7), and one for valence with the poles “unpleasant” (1) and “pleasant” (7). In addition to the words, the corresponding smiley pictures from the affective sliders by Betella and Verschure (2016) were shown at the end poles of the VASs.

Duration and POT judgments were provided in different halves of the experiment, with the order of the judgments counterbalanced across participants. In each half of the experiment, participants viewed and judged 12 pictures (three of each subset), each two times. The combination of durations (2, 4, and 6) and individual pictures was balanced across participants, and one picture was never shown twice with the same duration for the same participant. Within each experimental half, the pictures were presented in random order.

There were two attention checks during the experiment, one after the 10th picture in the first half of the experiment, and the other after the 14th picture in the second half of the experiment. In these trials, participants were presented with one of two “catch” pictures (presented for 4 s) and had to choose from four options which animal (horse, cow, rabbit, or pig) was shown on the picture (ID 624, Pig 1) or how many human beings (0, 1, 2 or 3) were shown on the picture (ID 392, Gazing 4).

Participants were instructed to view all the pictures without counting the seconds and to relax between picture presentations. In the POT condition, it was emphasized that the judgment concerned the feeling of how time passed while viewing the picture and not how long the picture was. For emotional ratings (arousal and valence), participants were asked to indicate the affective state evoked by viewing the respective picture.

Before the two experimental blocks, there was one practice trial, in which participants viewed an additional picture (ID 133, Cat 4) and provided the respective temporal judgments and the two emotional ratings (arousal and valence). Each experimental session lasted about 30 min.

## Results

All statistical analyzes were performed in R (Version 4.3.2; R Core Team, 2023). The R package *afex* (Singmann et al., 2023) was used for linear mixed-effects model (LMM) analyzes, and the packages *ggplot2* (Wickham, 2016) and *emmeans* (Lenth, 2023) were used to create figures.

### Arousal and valence ratings

**Fig. 1:**
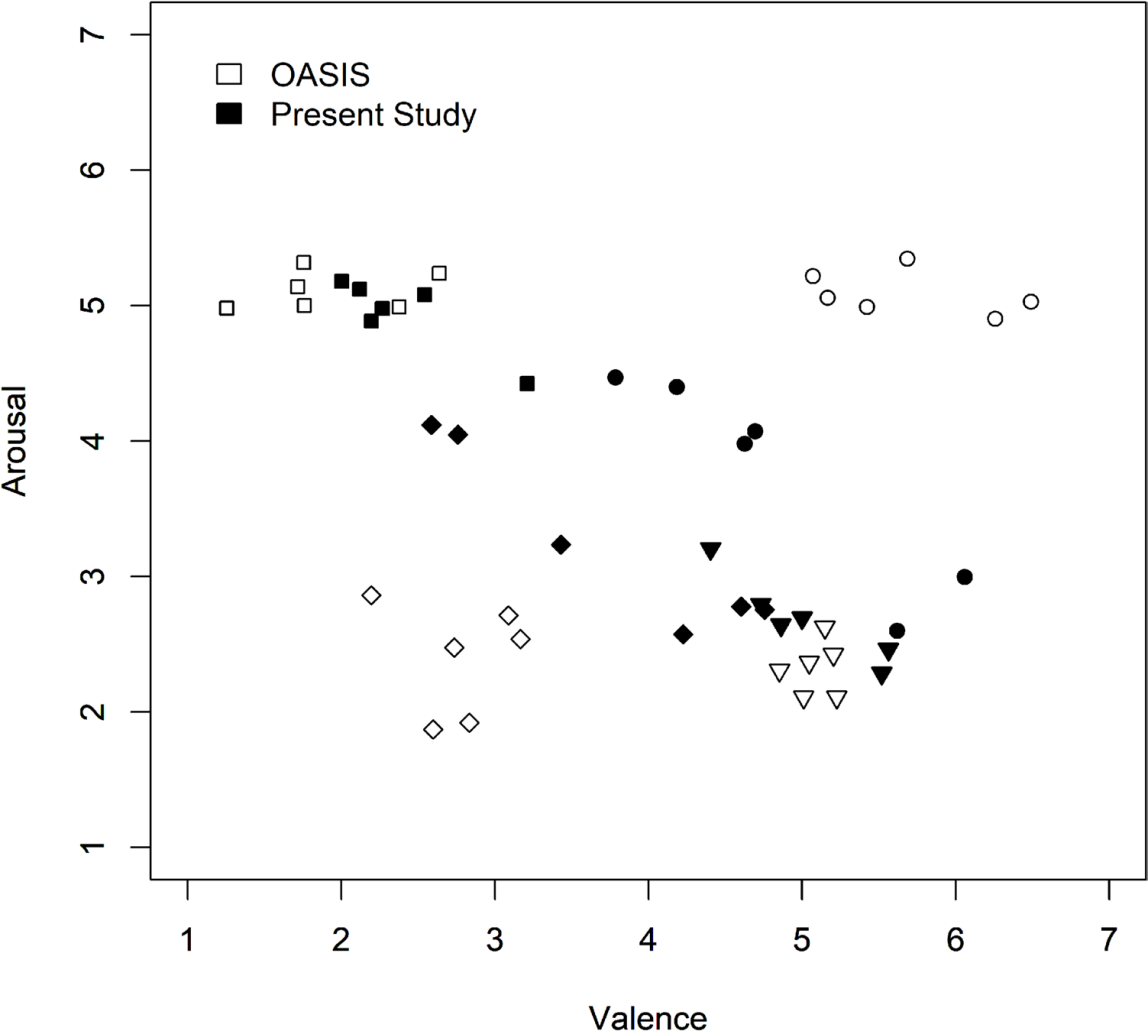
Mean arousal and valence ratings from OASIS and the present study. Symbols indicate the four different picture subsets included in the present study (circle: high/high; square: high/low; diamond: low/low; triangle: low/high)

The mean arousal and valence ratings for the four picture subsets were (with corresponding means of OASIS): high arousal/high valence: 3.75 (5.09), 4.83 (5.68), high arousal/low valence: 4.95 (5.11), 2.39 (1.92), low arousal/high valence: 2.68 (2.32), 5.01 (5.08) and low arousal/low valence: 3.25 (2.40), 3.73 (2.77). In particular, the two subsets high arousal/high valence and low arousal/low valence deviated from the OASIS ratings. Compared with the OASIS ratings, they showed a tendency towards medium ratings (see Fig. 1). Accordingly, the mean arousal and valence ratings provided by the present participants were strongly negatively correlated, *r* = −.86, *p* <.001, whereas the arousal and valence ratings from OASIS for the current picture sample were uncorrelated, *r* = −.05, *p* =.800. The current ratings, however, were still positively correlated with the OASIS ratings (arousal: *r* =.49, *p* <.001; valence: *r* =.65, *p* <.001). Because of these differences between the current ratings and those of OASIS, temporal judgments were analyzed in two different ways, one with the factorial design based on the OASIS ratings and one with the individual ratings obtained from the participants of the present study.

### Effects of arousal and valence (OASIS) on duration and POT judgments

**Fig. 2:**
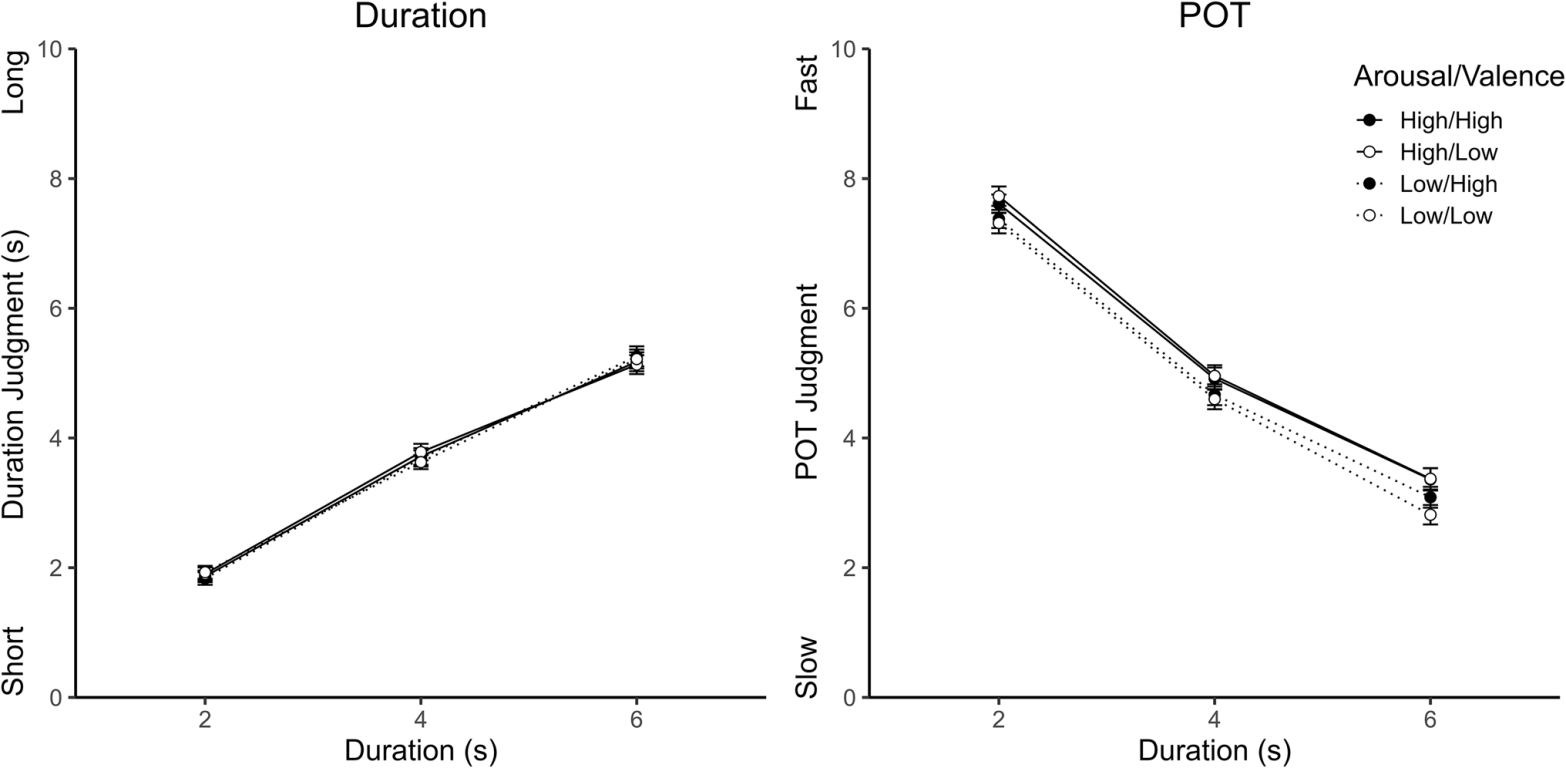
Mean duration and passage of time (POT) judgments as a function of arousal, valence and duration. Error bars represent ± 1 within-subject standard errors (Morey, 2008)

The results regarding the four picture subsets based on OASIS ratings are depicted in Fig. 2. Since duration and POT judgments showed inverse trends across durations, POT judgments were inverted for further analyzes (0 = *very fast*, 10 = *very slowly*; see also Bratzke, 2025), but descriptive statistics are still reported based on noninverted POT judgments. An ANOVA with the within-subject factors judgment type (duration vs. POT), duration, arousal (high vs. low), and valence (high vs. low) on temporal judgments showed significant main effects of judgment type, *F*(1, 86) = 96.11, *p* <.001, η_p_^2^ =.53, duration, *F*(1, 86) = 873.11, *p* <.001, η_p_^2^ =.91, and arousal, *F*(1, 86) = 12.94, *p* <.001, η_p_^2^ =.13. There was no significant main effect of valence, *F*(1, 86) = 0.22, *p* =.642, η_p_^2^ <.01. Importantly, there were also significant interactions between judgment type and duration, *F*(1, 86) = 35.92, *p* <.001, η_p_^2^ =.29, and between judgment type and arousal, *F*(1, 86) = 14.08, *p* <.001, η_p_^2^ =.14, but no significant interaction between judgment type and valence, *F*(1, 86) = 0.10, *p* =.754, η_p_^2^ <.01. Separate post hoc ANOVAs for duration and POT judgments showed that the effect of arousal was significant for POT judgments, *F*(1, 86) = 17.47, *p* <.001, η_p_^2^ =.17, but not for duration judgments, *F*(1, 86) = 0.01, *p* =.931, η_p_^2^ <.01. POT judgments were higher (which means faster) for high arousal pictures (5.32) than for low arousal pictures (4.98), whereas this difference was virtually zero for duration judgments (3.60 vs. 3.59).

### Effects of current arousal and valence ratings on duration and POT judgments

**Fig. 3:**
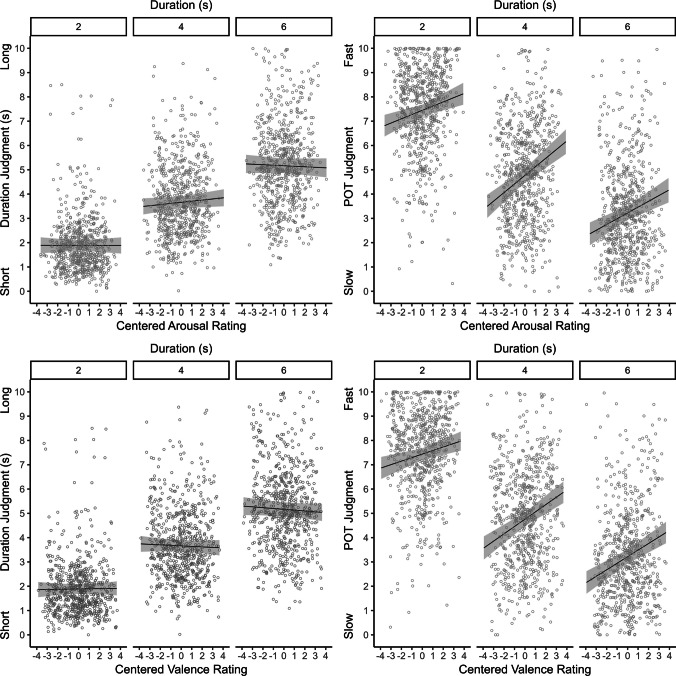
Linear mixed-effects model predictions for duration and POT judgments as a function of duration and affective ratings (upper panels: arousal; lower panels: valence). Lines represent model predictions, grey areas represent confidence intervals, and grey circles represent current temporal judgments (duration and POT). Arousal and valence ratings are centered for each participant

To analyze the effects of arousal and valence on temporal judgments based on the subjective ratings obtained from the present participants, LMM analyzes were conducted. In an overall model, temporal judgments (duration and inverted POT judgments) were predicted by arousal and valence ratings (both centered for each participant; see Brauer & Curtin, 2018), judgment type (duration vs. POT) and duration as fixed effects, with random intercepts per participant and random slopes per participant and judgment type.^2^ This analysis showed significant effects of arousal and valence ratings [arousal: *F*(1, 4012.78) = 48.14, *p* <.001; valence: *F*(1, 4001.51) = 62.46, *p* <.001] and interactions between judgment type and arousal rating, *F*(1, 3998.43) = 47.82, *p* <.001, and between judgment type and valence rating, *F*(1, 3994.07) = 41.04, *p* <.001. Separate post hoc LMMs for duration and POT judgments (including random intercepts per participants and random slopes for duration per participant) showed that duration judgments were significantly affected only by duration,* F*(2, 110.80) = 344.99, *p* <.001 (all other *p* values >.060). In contrast, POT judgments were affected by duration, *F*(2, 113.33) = 340.24, *p* <.001, and also by both affective ratings [arousal: *F*(1, 1929.41) = 68.69, *p* <.001; valence: *F*(1, 1910.01) = 75.33, *p* <.001]. For POT judgments, there was also a significant interaction between valence rating and duration, *F*(1, 1910.93) = 3.09, *p* =.046 (all other *p* values >.053). As can be seen in Fig. 3, both higher arousal and higher valence lead to faster POT judgments.

## Discussion

The present study investigated the effects of arousal and valence of emotional pictures on the perception of duration and the experience of the POT. While there is considerable literature on the effects of emotional stimuli on duration perception, studies on their effects on the POT experience are yet very rare. To my knowledge, the present study is the first to consider both effects within the same study. In contrast to previous studies, variations in arousal or valence did not affect duration perception. However, both higher arousal and higher valence increased the speed of the POT. Furthermore, the present study replicates the previous finding that the subjective POT slows down with increasing duration (Bratzke, 2025; Jording et al., 2022; Martinelli & Droit-Volet, 2022b). Most importantly, the present dissociation between duration and POT judgments with regard to the influence of emotional pictures suggests that these temporal experiences are not just two sides of the same coin, or that one is derived from the other.

The absence of arousal and valence effects on duration judgments in the present study is striking, as it contrasts with previous studies using similar emotional stimuli (Angrilli et al., 1997; Noulhiane et al., 2007; Van Volkinburg & Balsam, 2014). Notably, Angrilli et al. did not observe main effects of arousal and valence but an interaction between the two dimensions, which, however, was also not observed in the present study. In this regard, it is important to note that, although the four picture subsets included in the present study were carefully selected to form distinct clusters regarding arousal and valence ratings, pictures with extreme contents were not included and therefore not the whole spectrum of possible arousal and valence values was tested. Additionally, the arousal and valence ratings provided by the participants in the present study differed from the OASIS ratings (Kurdi et al., 2017), with generally smaller differences of the mean arousal and valence ratings between the different picture subsets and a strong negative correlation between mean arousal and valence ratings.^3^ Whether these differences are due to the different rating contexts,^4^ habituation, historical or cultural effects, or reflect sample-specific differences is unclear. Nevertheless, it is possible that the differences between the high and low arousal pictures were not large enough to influence duration judgments. Another factor that might modulate the influence of arousal (and perhaps also of valence) on duration judgments is stimulus duration, as Angrilli et al. observed an effect of arousal only for short (2 s) durations (see also Noulhiane et al., 2007). This factor, however, cannot account for the absence of arousal and valence effects in the present study, as the same stimulus durations as in Angrilli et al. were used, including the 2 s condition. It should be noted that the present study was very similar to previous studies not only regarding the tested durations (2–6 s; Angrilli et al., 1997; Noulhiane et al., 2007) but also regarding the method to assess duration judgments (VAS; Angrilli et al., 1997). Furthermore, the sample size of the present study (*N* = 87) was larger than in previous studies (Angrilli et al., 1997: *N* = 51; Noulhiane et al., 2007: *N* = 17–24) so that the statistical power was probably also higher. Thus, it seems likely that, due to the limited selection of emotional pictures included in the present study, the variations in arousal and valence were not large enough to affect duration judgments through changes in the rate of the internal pacemaker rate or attentional mechanisms.

Importantly, although arousal and valence did not affect duration judgments, they clearly affected POT judgments, with an acceleration of the POT for high-arousal and high-valence stimuli. However, as there was a strong negative correlation between current arousal and valence ratings, it is unclear whether these effects reflect the influence of one affective dimension (arousal or valence) or both. While it is difficult to determine which affective dimension ultimately caused the effect on the POT experience, the valence effect on POT judgments observed in the present study is consistent with previous findings by Martinelli and Droit-Volet (2022b) and with the common assumption that hedonic valence influences the POT experience (e.g., Droit-Volet & Martinelli, 2023; Droit-Volet & Wearden, 2016; Droit-Volet et al., 2017; Wittmann et al., 2015). However, in contrast to the present study, Martinelli and Droit-Volet used videos of sequentially presented static pictures (with a total duration of 30 to 108 s) instead of short static pictures “to maintain the emotion induced in the case of long durations of several minutes” (p. 525). The present results suggest that such long durations are not necessary for the valence of emotional pictures to influence the POT experience.

The present dissociation between duration and POT judgments is reminiscent of a similar dissociation observed by Droit-Volet and Wearden (2016) and Droit-Volet et al. (2017) using the experience sampling method. In these studies, participants were alerted several times per day during their normal daily lives via their mobile phones and had to estimate durations and report their POT experience and their affective state at the time of the alert. In both studies, there was no significant relation between duration and POT judgments for rather short durations (500–1,500 ms in Droit-Volet & Wearden, 2016, and 3–33 s in Droit-Volet et al., 2017). Additionally, in the study by Droit-Volet et al., higher arousal was associated with an accelerated POT, but arousal did not affect duration judgments (for a similar effect of self-reported arousal on POT judgments, see Droit-Volet & Wearden, 2015). Furthermore, there also appear to be other nonaffective factors, such as event density, which also have a stronger influence on the experience of the POT than on the perception of duration (Jording et al., 2022; Lamprou-Kokolaki et al., 2024).

POT judgments might reflect actual experiences of the POT (e.g., due to introspective analysis of the effect of internal and external contextual changes on internal states; Martinelli & Droit-Volet, 2022b), or retrospective inferences (e.g. in response to an external cue like a clock reading or based on mental concepts of time experience; Wearden, 2015), and it is often difficult to determine the basis on which POT judgments are made (see also Jording et al., 2022). Martinelli and Droit-Volet (2022b) suggested that POT judgments can be based on duration perception, when changes in stimulus duration are the most salient information. The present affective effects on POT judgments clearly cannot be based on duration perception, as duration judgments were not affected in a similar way by the emotional pictures. It therefore seems more likely that these effects reflect changes in the actual experience of the POT, which can be attributed to the influence of the emotional pictures on the internal (emotional) state while viewing the pictures.

There is, however, an alternative explanation for the present affective effects on POT judgments, related to the specific picture content. Lamprou-Kokolaki et al. (2024) observed that POT judgments scaled with event density (i.e., the number of events in a given duration), suggesting that the overall complexity of a scene and the rate of events are important factors for the subjective POT (see also Jording et al., 2022). As Carrozzo et al. (2010) have shown animacy effects on time perception for moving as well as for still characters, and animacy probably implies a potentially higher rate of change, animacy might be a confounding factor for the affective effects on POT judgments in the present study. In fact, seven of the 12 pictures (58%) from the two high-arousal sets showed animate objects (humans or animals), whereas this was the case only for two of the low-arousal subsets (17%). Similarly, for the valence dimension the proportion of animate to inanimate contents was higher for the high- (50% animate) than for the low-valence (25% animate) picture subsets.^5^ Thus, it is possible that animacy and its presumably associated higher activity or rate of change contributed to the present findings. A similar confound between animacy and arousal also exists in memory research, where animate entities are often better remembered than inanimate ones (e.g., Nairne et al., 2013). This animacy advantage, however, can also be observed when the possible confounding effect of arousal is controlled for (Meinhardt et al., 2018; Popp & Serra, 2018). It remains to be seen whether similar differential contributions of animacy and arousal can also be demonstrated for time perception.

In conclusion, the present study showed differential effects of arousal and valence of emotional pictures on duration and POT judgments. While duration judgments were not affected by the two affective dimensions, this was the case for POT judgments, with both higher arousal and valence leading to an accelerated POT. In addition to the observed dissociation between duration perception and the experience of the POT, the present study highlights the importance of collecting and analyzing current affective ratings even when using standardized emotional pictures. As these ratings might differ significantly from the norm values of the standardized picture sets, relying solely on the latter could lead to incorrect conclusions.

## Data Availability

The data generated during and/or analyzed during the current study are available via OSF: https://osf.io/4ekxh

## Footnotes

1 Angrilli et al. (1997) used temporal reproduction and duration estimation via a visual analogue scale in different groups of participants. However, the interaction effect of valence and arousal on duration estimates was independent of the timing method.

2 The random effect structures of the LMMs were adjusted iteratively to prevent the models from becoming singular.

3 Although Angrilli et al. (1997) also assessed arousal and valence ratings, they did not report the mean ratings, so that an evaluation of their study regarding this issue is not possible.

4 In Kurdi et al. (2017), participants rated the pictures included in the present study in the context of even more extreme, but also neutral pictures. Furthermore, each participant rated only one affective dimension (arousal or valence), and participants who rated the arousal dimension were explained the theoretically orthogonal nature of the two dimensions.

5 These proportions of animate vs. inanimate contents are consistent with Kurdi et al.’s (2017) analysis of OASIS regarding the relationship between picture categories (animals, people, scenes, and objects) and the two affective ratings.
